# Supplementary material for: Complex‐centric proteome profiling by SEC‐SWATH‐MS
Source: Mol Syst Biol. 2019 Jan 14;15(1):e8438. doi: 10.15252/msb.20188438 (PMC6346213; doi:10.15252/msb.20188438)
Supplement: Supplementary file 6 — Dataset EV5 [file MSB-15-e8438-s006.zip › feature_plots_corum/650-1.pdf]

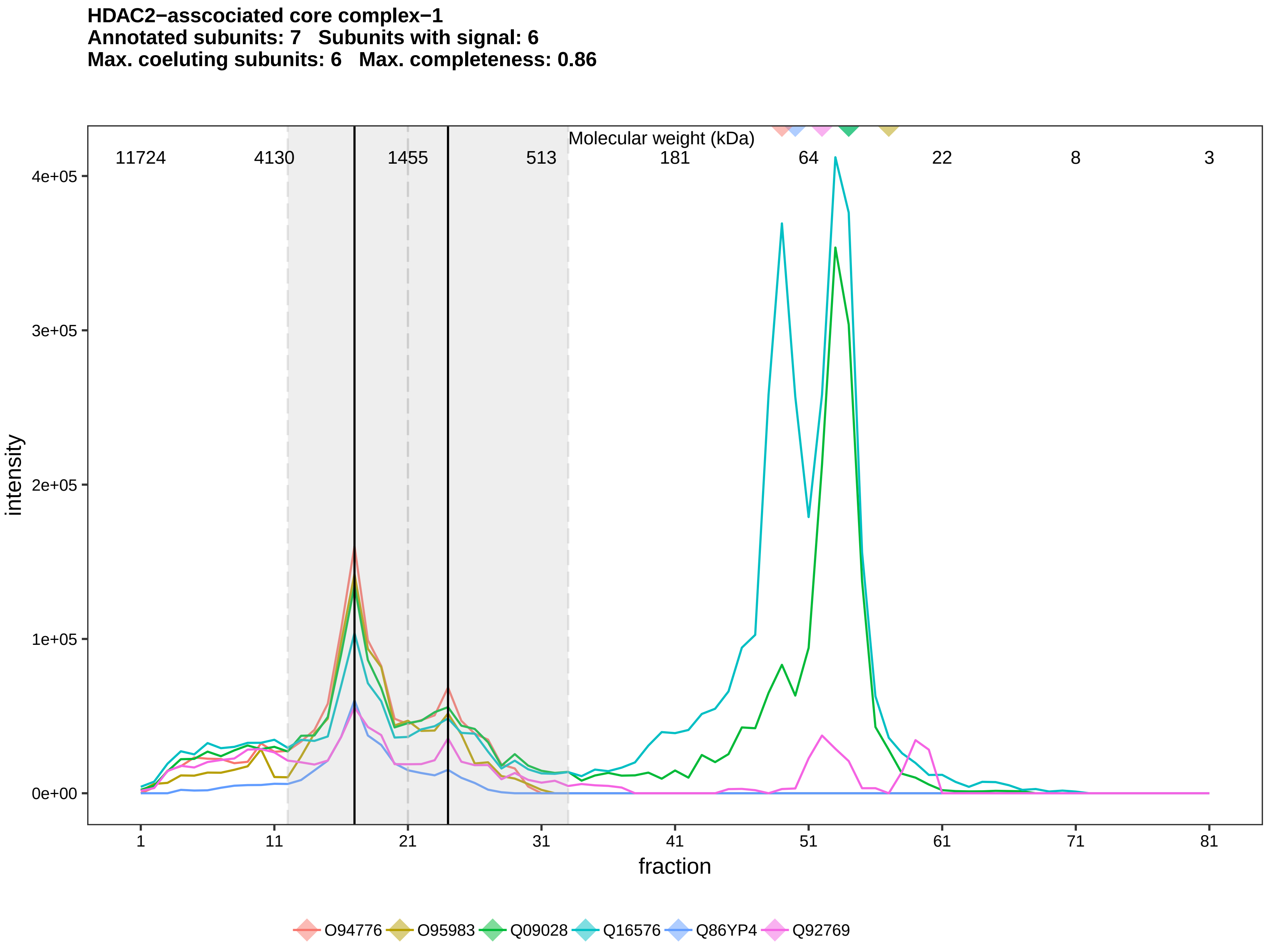
HDAC2−asscociated core complex−1
Annotated subunits: 7   Subunits with signal: 6
Max. coeluting subunits: 6   Max. completeness: 0.86
Molecular weight (kDa)
11724
4130
1455
513
181
64
22
8
3
4e+05
3e+05
2e+05
1e+05
0e+00
intensity
1
11
21
31
41
51
61
71
81
fraction
O94776
O95983
Q09028
Q16576
Q86YP4
Q92769
